# Effects of MUL1 and PARKIN on the circadian clock, brain and behaviour in *Drosophila* Parkinson’s disease models

**DOI:** 10.1186/s12868-019-0506-8

**Published:** 2019-05-28

**Authors:** Bartosz Doktór, Milena Damulewicz, Elżbieta Pyza

**Affiliations:** Department of Cell Biology and Imaging, Institute of Zoology and Biomedical Research, Jagiellonian University, Kraków, Poland

**Keywords:** Mitochondrial ligases, Clock genes, Clock neurons, ROS, SOD1, Autophagy, Locomotor activity rhythm, Sleep

## Abstract

**Background:**

Mutants which carry mutations in genes encoding mitochondrial ligases MUL1 and PARKIN are convenient *Drosophila* models of Parkinson’s disease (PD). In several studies it has been shown that in Parkinson’s disease sleep disturbance occurs, which may be the result of a disturbed circadian clock.

**Results:**

We found that the ROS level was higher, while the anti-oxidant enzyme SOD1 level was lower in *mul1*^*A6*^ and *park*^*1*^ mutants than in the *white* mutant used as a control. Moreover, mutations of both ligases affected circadian rhythms and the clock. The expression of clock genes *per*, *tim* and *clock* and the level of PER protein were changed in the mutants. Moreover, expression of ATG5, an autophagy protein also involved in circadian rhythm regulation, was decreased in the brain and in PDF-immunoreactive large ventral lateral clock neurons. The observed changes in the molecular clock resulted in a longer period of locomotor activity rhythm, increased total activity and shorter sleep at night. Finally, the lack of both ligases led to decreased longevity and climbing ability of the flies.

**Conclusions:**

All of the changes observed in the brains of these *Drosophila* models of PD, in which mitochondrial ligases MUL1 and PARKIN do not function, may explain the mechanisms of some neurological and behavioural symptoms of PD.

**Electronic supplementary material:**

The online version of this article (10.1186/s12868-019-0506-8) contains supplementary material, which is available to authorized users.

## Introduction

Mitochondria are important organelles in the metabolism of all cells, particularly in neurons because of their high energy demand [1]. As a result, several neurodegenerative diseases in which changes in mitochondrial structure and function lead to cell death [2]. One of these diseases is Parkinson’s disease (PD), which can be caused by exposure to neurotoxins and/or by several gene mutations. These mutations include mutations in genes encoding PINK1, a mitochondrial kinase, and PARKIN, an E3 ubiquitin ligase, which leads to the autosomal recessive form of PD [3, 4]. These proteins regulate the function and morphology of mitochondria and promote mitophagy [5, 6]. Dysfunctional and damaged mitochondria lose their membrane potential, leading to activation and accumulation of PARKIN and degradation of the whole organelle [7, 8]. In addition, there is another pathway that promotes mitophagy that acts in parallel to PINK1/PARKIN. This pathway involves mitochondrial ubiquitin ligase 1 (MUL1), which is responsible for mitochondrial integrity, fusion–fission processes, mitophagy and SUMOylation. Mutations in *mul1* lead to typical PD symptoms, similar to those observed in *pink1*/*park* mutants [9]. In addition to molecular symptoms such as those observerd in *mul1* and *park* mutants, Parkinson’s disease are also characterized by other motor and non-motor symptoms. Main motor disorders are bradykinesia and tremor, while non-motor disorders include pain, cognitive deficits, depression and sleep problems due to restless legs syndrome, REM sleep behaviour disorder (RBD) and excessive daytime sleepiness (EDS) or insomnia [10]. Sleep fragmentation and reduced sleep efficiency have an impact on patient quality of life and may accelerate the development of PD. Non-motor symptoms have also been observed in circadian rhythms of core body temperature and blood pressure. Because the circadian clock controls many of the abovementioned processes [11–13], the non-motor symptoms of PD might be a consequence of circadian clock malfunction and not a direct cause of the disease.

The *Drosophila* central clock (pacemaker) is composed of 150 neurons in the brain. These neurons are grouped into several clusters: large ventral lateral neurons (l-LNvs), small ventral lateral neurons (s-LNvs), lateral dorsal neurons (LNds), dorsal neurons (DN1-DN3) and posterior lateral neurons (LPNs) [14]. All l-LNvs and four of the five s-LNs are immunoreactive to pigment-dispersing factor (PDF), a main neurotransmitter of the clock. The molecular mechanism of the fruit fly clock is based on the cyclic expression of several clock genes and their proteins. The core clock genes include *period* (*per)*, *timeless* (*tim), clock* (*clk*) and *cycle* (*cyc*) [15]. *Per* and *tim* expression is activated at the end of the day and at the beginning of the night by CLK/CYC transcription factors, acting as heterodimers. PER and TIM are synthesized at the end of the night, form heterodimers and, by entering the nucleus, inhibit the activity of CLK and CYC and the transcription of their own genes. This negative feedback loop is the main mechanism of the clock.

In the present study, we examined whether mutations of *mul1* and *park* genes affect the molecular mechanism of the circadian clock and clock neurons, which may lead to changes in behavioural circadian rhythms and sleep disturbance. We studied whether both mutations affect cell protective mechanisms, synthesis of antioxidant proteins and autophagy. To evaluate the phenotypes associated with *mul1*^*A6*^ and *park*^*1*^ mutants, we also examined the longevity of the flies. We used *Drosophila melanogaster* since similar mechanisms and phenotypes of some human diseases have been described in this species [5, 6]. In addition, *Drosophila* is a primary model used in neuroscience and the study of circadian rhythms and clocks.

## Materials and methods

### Animals

The following strains were used for the experiments: *park*^*1*^ (null mutation of the *park* gene encoding the mitochondrial ligase PARKIN, Bloomington Drosophila Stock Centre) [16], *mul1*^*A6*^ (null mutation of the *mul1* gene encoding the mitochondrial ligase MUL1, kindly donated by Dr. Ming Guo, Brain Research Institute, USA) [9] and *w*^*1118*^ (null mutation of the *white* gene encoding ABC transporter, Bloomington Drosophila Stock Centre, which was used as controls because of the *white* genetic background of mutants *mul1*^*A6*^ and *park*^*1*^) [17]. Flies were maintained on standard yeast-cornmeal-agar medium at 25 ± 1 °C under a day/night cycle, LD 12:12 (12 h of light and 12 h of darkness).

### Locomotor activity rhythm and sleep analyses

One- to two-day-old males (N = 32) were transferred to small glass tubes containing sugar-agar food medium. Vials were placed in DAMS monitors (Drosophila Activity Monitoring System, TriKinetics) and inside an incubator (25 °C). Monitors were equipped with infrared sensors that recorded the activity of the flies inside the vials every 5 min. For the first 5 days, monitors were held in LD 12:12 conditions, followed by constant darkness (DD) for the next 6 days. The results from the second day of locomotor activity recording were analysed to estimate the total activity and duration of sleep during the day and during the night (Microsoft Excel plugin, BeFly kindly donated by Dr. E. Green, University of Leicester, [18] and Python 22 (http://www.python.org/). Sleep in flies was defined as the time in which they did not change their position for at least 5 min. The experiment was repeated three times. In LD 12:12 and DD, the rhythm of locomotor activity was also examined, and the period of the circadian rhythm was estimated in DD.

### Whole-brain immunohistochemistry

Seven-day-old males were fixed at ZT1 in 4% paraformaldehyde in 0.2% PBT for 3 h at 4 °C. Isolated brains were washed six times in PBS for 5 min each time. Next, they were incubated in 5% normal goat serum (NGS) and 0.5% bovine serum albumin (BSA) for 30 min at room temperature. Subsequently, brains were incubated overnight with a mouse primary antibody targeting PDF (1:1000, Developmental Studies Hybridoma Bank) or a rabbit antibody targeting ATG5, an autophagy protein (1:500, Abcam). Afterwards, brains were washed six times in 0.2% PBT for 5 min each time and incubated overnight at 4 °C with secondary goat anti-mouse Cy3-conjugated (1:500, Jackson ImmunoResearch) or goat anti-rabbit Alexa 488-conjugated (1:1000, MolecularProbes) antibodies, depending on the primary antibodies. Finally, brains were washed four times in 0.2% PBT and twice in PBS and mounted in Vectashield medium (Additional file 1: Table S1).

### Quantification of immunolabelling

To measure the fluorescence intensity of ATG5 protein in the large ventral lateral neurons (l-LNvs), we used confocal microscopy. We identified cell bodies of l-LNvs using the anti-PDF antibody, and we scanned the same cell for labelling with the anti-ATG5 antibody. We selected all l-LNv cell bodies and measured the fluorescence intensity of the ATG5 protein. Images were collected with a Zeiss Meta 510 Laser Scanning Microscope (Additional file 2: Table S2).

### ROS measurements

To measure whether ROS levels were increased in *mul1*^*A6*^ and *park*^*1*^ mutants, 7-day-old males (N = 10) of mutants and controls were decapitated at ZT0. Brains were isolated and washed twice in PBS for 10 min. Next, the tissue was incubated with MitoSOX (ThermoFisher) for 10 min and mounted in Vectashield medium. Images were collected with a Zeiss Meta 510 Laser Scanning Microscope. The method has been described by Scialò et al. [19].

### Western blot

Seven-day-old males (N = 30) were frozen in liquid nitrogen at four time points (ZT1, ZT4, ZT13, ZT16) and decapitated. Heads were homogenized by sonication in 30 µl of Laemmli buffer with a protease inhibitor (Boehringer, Mannheim), left for 30 min at 4 °C and frozen at − 20 °C. Homogenates were centrifuged at 13,200 rpm for 1 h at 4 °C. Supernatants were collected and denatured at 85 °C for 5 min. Total protein levels were measured using a Quant-iT Protein Assay Kit and Qubit fluorometer (Invitrogen). Afterwards, 20 µg of protein from each supernatant was subjected to electrophoresis (NuPAGE 4–12% bis–Tris gels, Invitrogen) at 165 V for 40 min and then blotted by electrotransfer onto a PVDF membrane (Invitrogen) at 30 V for 60 min. The membrane was blocked in 5% non-fat dry milk in PBS with 0.1% Tween 20 (TBS) for 1 h at 4 °C and incubated with a mix of primary antibodies: anti-ATG5 (1:1000), anti-PER (1:10,000, kindly donated by Dr. Ralph Stanewsky, University of Münster, Germany) or anti-SOD1 (1:5000, Abgent) and anti-α tubulin (1:20,000, Developmental Studies Hybridoma Bank) in 1% BSA in 0.1% TBS overnight at 4 °C. Next, the membrane was washed 5 times in 0.1% TBS for 10 min each time and incubated with secondary antibodies conjugated to HRP (1: 10,000, Abcam) in 0.1% TBS with 1% BSA for 1 h at room temperature. After incubation, the membrane was washed 5 times in 0.1% TBS and immunodetected with an ECL detection system (Perkin Elmer). Densitometric analysis of Western blot was performed using ImageJ. The experiment was repeated three times (Additional file 3: Table S3).

### qPCR

Seven-day-old males (N = 20) were decapitated at ZT1, ZT4, ZT13 or ZT16 in LD 12:12. Heads were fixed in 100% ethanol for 2 h, and brains were isolated. Total RNA was isolated using TriReagent (MRC Inc.). Total RNA (5 μg) was used for reverse transcription [High-Capacity cDNA Reverse Transcription Kit (ThermoFisher)] according to the manufacturer’s protocol. A total of 1000 ng cDNA (diluted 1:10) was used for quantitative PCR. Each experiment was repeated three times. The expression of the following clock genes *per, tim* and *clock* was examined using TaqMan (ThermoFisher). *per* (Dm01843683), *tim* (Dm01814242) *clock* (Dm01795381) and reference gene *rpl32* (Dm02151827) probes were also obtained from ThermoFisher. The reaction was performed using the StepOnePlus Real-Time PCR System (ThermoFisher). Data were collected as raw CT values and analysed using the 2 − ΔΔCT method. Gene expression was normalized on an arbitrary scale normalized to control (Additional file4: Table S4).

### Lifespan assay

One-day-old males of each strain were placed into vials containing the standard food medium (20 flies per vial). Every 3–4 days, flies were transferred to new vials with fresh food, and the number of dead flies was counted. The experiment was repeated three times.

### Statistics

The statistical analyses were performed using GraphPad Prism 6. Normal distribution of data was examined, and statistical tests were chosen accordingly. For lifespan analysis, the Kaplan–Meier test was used. The Wilcoxon–Mann–Whitney and Kruskal–Wallis tests were performed to assess differences in sleep and total activity. The results obtained from analysis of period of the circadian rhythm of locomotor activity, climbing assay, Western Blot, qPCR, ROS measurements and the fluorescent intensity associated with ATG5 level were analysed using one-way ANOVA and Tukey’s test.

## Results

### Effects of *mul1* and *park* mutations on lifespan and behaviour

**Fig. 1:**
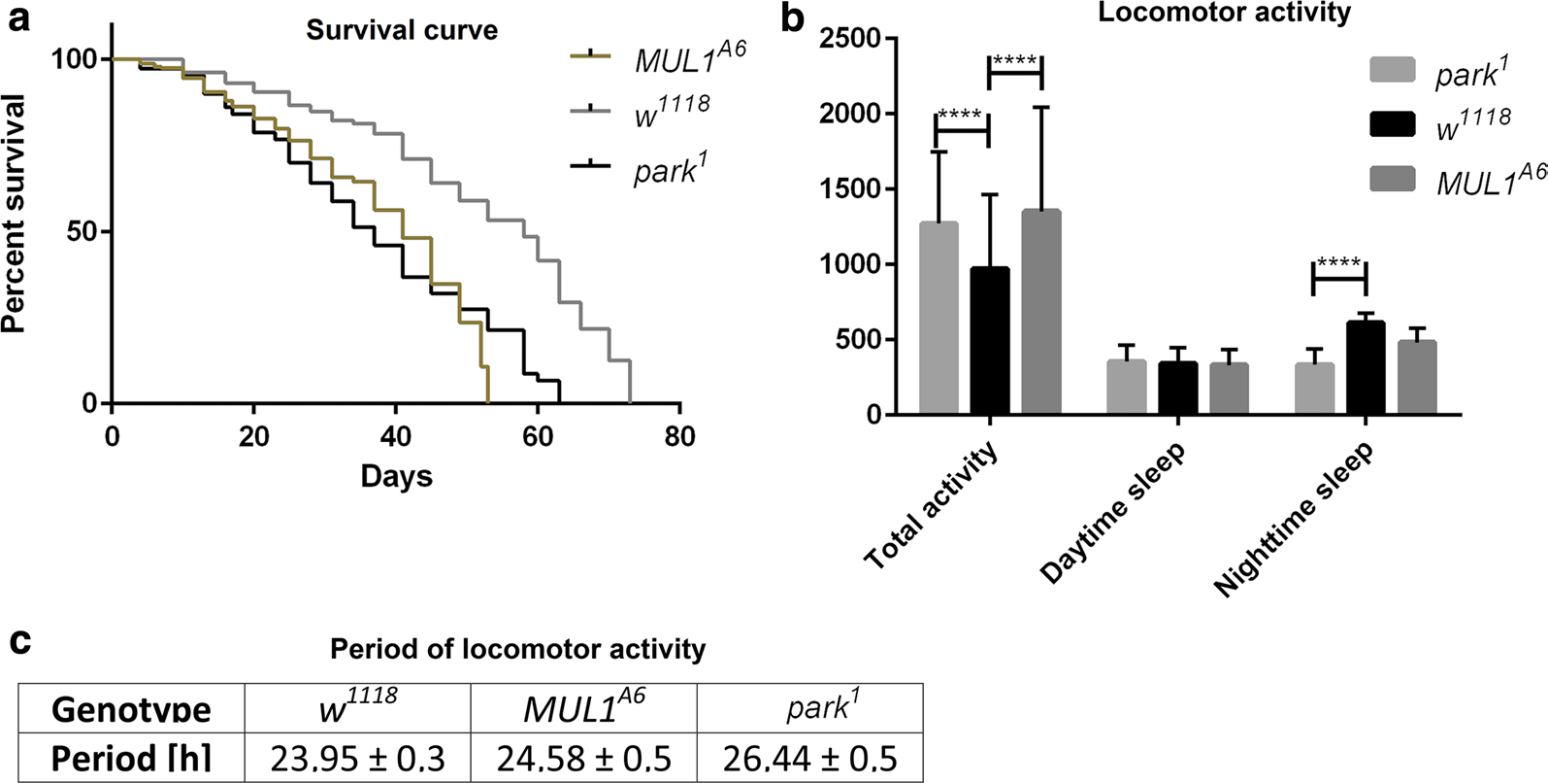
Effects of *mul1* and *park* mutations on lifespan and the circadian rhythm of locomotor activity. **a** Kaplan–Meier survival curve. Dead flies were counted every 3 days. Statistically significant differences were detected between *white* control and *mul1* (p < 0.05), and also between *white* and *park* mutants (p < 0.05). N of *w*^*1118*^ = 231, N of *park*^*1*^ = 150, N of *mul1*^*A6*^ = 233. **b** Total activity and sleep duration during the day and at night in LD 12:12. The Y-axis shows time in minutes when flies were active or their sleep time (means ± SD) (four asterisks indicates p < 0.01). N of *w*^*1118*^ = 25, N of *park*^*1*^ = 29, N of *mul1*^*A6*^ = 21. **c** Period of locomotor activity rhythm in all studied genotypes (means ± SD). Statistically significant differences were detected between *white* control and *mul1* (p < 0.05), and also between *white* and *park* mutants (p < 0.05). N of *w*^*1118*^ = 31, N of *park*^*1*^ = 47, N of *mul1*^*A6*^ = 45

The lifespan analysis of *mul1*^*A6*^ and *park*^*1*^ mutants showed that both mutations significantly reduced longevity up to 30% and 25% in *mul1*^*A6*^ and *park*^*1*^, respectively (Fig. 1a). *mul1*^*A6*^ and *park*^*1*^ flies had longer total activity times during 24 h (LD 12:12) (Fig. 1b) than controls. Although the total activity was increased, the daytime sleep duration was the same across genotypes. However, the sleep duration during the night was decreased only in *park*^*1*^ mutants compared to that in control. Moreover, the period of locomotor activity rhythm was lengthened to ~ 27 h in *park*^*1*^ mutants and ~ 25 h in *mul1*^*A6*^ mutants, in contrast to the approximately 24-h period in *white* mutants, which was statistically significant (Fig. 1c).

### The effect of *mul1* and *park* mutations on the circadian clock

**Fig. 2:**
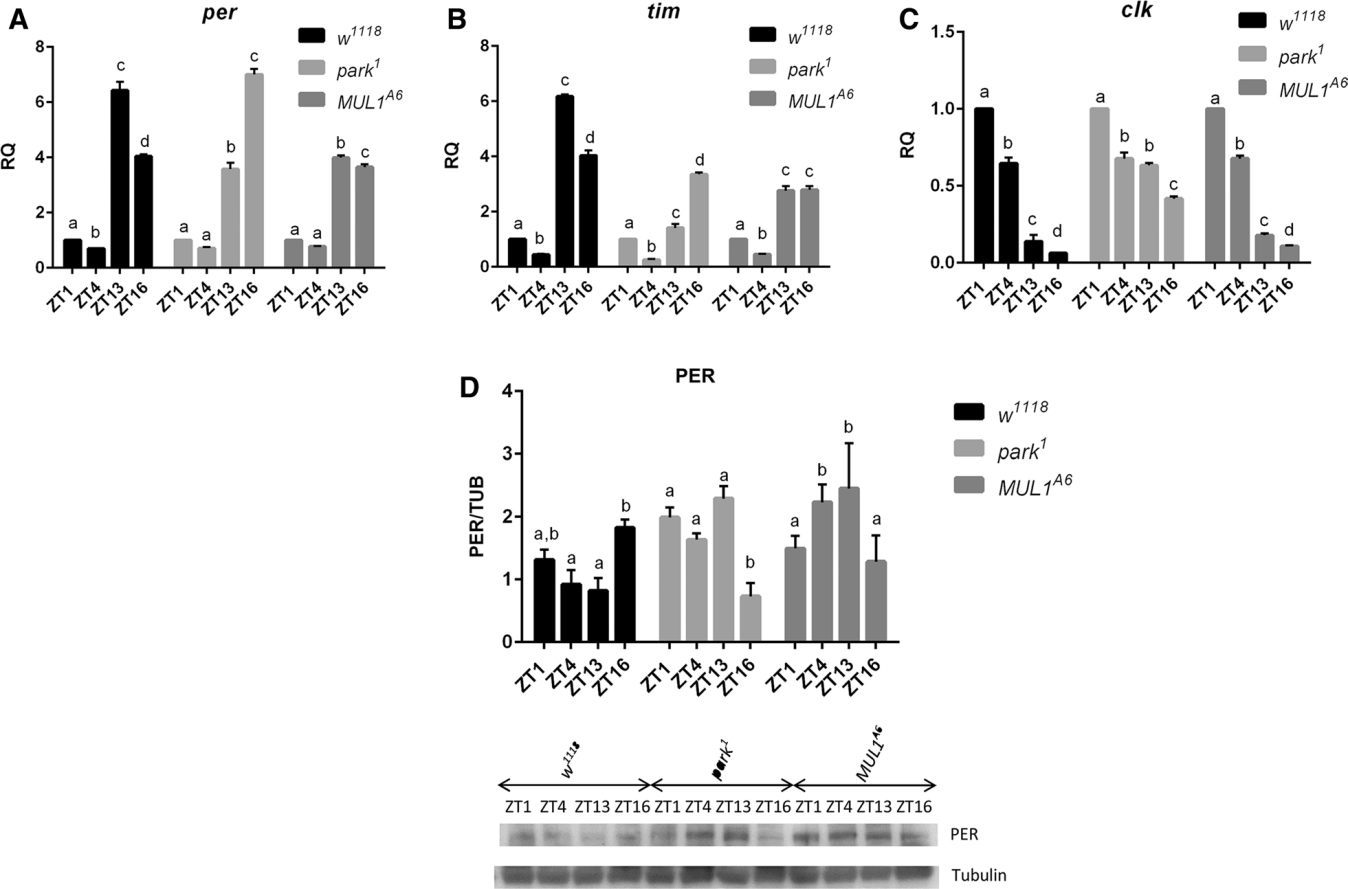
Effects of *mul1* and *park* mutations on the circadian clock. **A–C** Expression of selected clock genes in the *Drosophila* brain at four time points: ZT1, ZT4, ZT13, and ZT16 (means ± SD). Letters a, b, c and d indicate significant differences. Statistically significant differences are between *a* versus *b*, *a* versus *c*, *a* versus *d*, *b* versus *c*, *b* versus *d*, *c* versus *d* (p < 0.01) in each chart. N of *w*^*1118*^ = 60, N of *park*^*1*^ = 60, N of *mul1*^*A6*^ = 60. N of *w*^*1118*^ = 60, N of *park*^*1*^ = 60, N of *mul1*^*A6*^ = 60. **D** PER protein level in the *Drosophila* brain at four time points: ZT1, ZT4, ZT13, and ZT16 (means ± SD). Letters a, b, c and d indicate significant differences. Statistically significant differences are between *a* versus *b*, *a* versus *c*, *a* versus *d*, *b* versus *c*, *b* versus *d*, *c* versus *d* (p < 0.05). N of *w*^*1118*^ = 90, N of *park*^*1*^ = 90, N of *mul1*^*A6*^ = 90

Examination of clock gene expression showed that *mul1* and *park* mutations disrupted their normal expression during the day. In both *mul1*^*A6*^ and *park*^*1*^ mutants, the morning (ZT1) peak of *per* expression in the brain was broader than that in the *white* control flies (Fig. 2a). The peak of *per* mRNA expression in *park*^*1*^ mutants was at ZT16, while in the *white* control, the peak was at ZT13, and the expression of *per* was around 40% higher at this time point than the expression in *park*^*1*^ mutants. The *mul1*^*A6*^ mutants had around 35% smaller peak in *per* mRNA expression at ZT13 than *w*^*1118*^, but at ZT16, the *per* expression peak was similar to that in the control flies. Both *mul1*^*A6*^ and *park*^*1*^ mutants had reduced mRNA levels of *tim* at ZT13 (70% in *park*^*1*^ and 55% in *mul1*^*A6*^). In *park*^*1*^, the maximum *tim* expression was at ZT16, similar to peak *per* expression, but in *mul1*^*A6*^ mutants, the level of *tim* mRNA was the same at ZT13 and ZT16. The morning peak was the same in all genotypes studied (Fig. 2b). Daily oscillation of *clk* was only changed in *park*^*1*^ mutants, in which the peak at ZT13 was the same as that at ZT4, and the peak at ZT16 was five times higher than that in the *white* controls (Fig. 2c). Analysis of PER in the whole *Drosophila* brain showed that, in *park*^*1*^ mutants, PER protein expression did not differ among the ZT1, ZT4 and ZT13 time points, but at ZT16, PER protein expression was lower than the expression in *white* controls, in which the highest PER abundance was observed at ZT16. The level of PER at ZT1, ZT4 and ZT13 was 20, 25 and 60% higher, respectively, then the level in *w*^*1118*^ flies. *mul1*^*A6*^ mutants showed a similar level of PER at ZT1 as control flies, but at ZT4 and ZT13, the level of PER was 50% and 65% higher, respectively. PER protein expression was at its lowest at ZT16 (Fig. 2d, e).

### The effect of *mul1* and *park* mutations on ROS and endogenic antioxidants

**Fig. 3:**
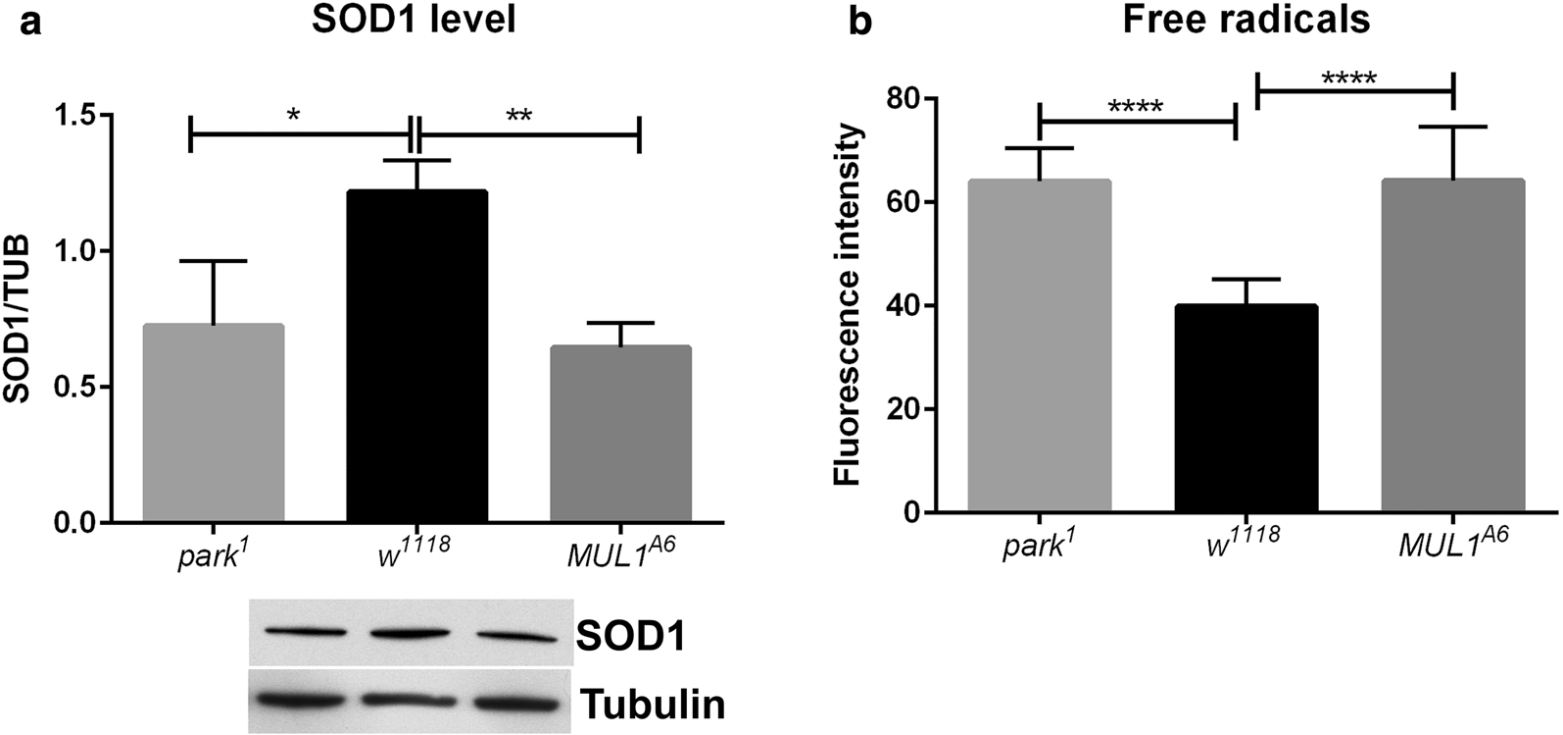
Effects of *mul1* and *park* mutations on SOD1 and ROS levels. **a** SOD1 level in the *Drosophila* brain (means ± SD). Statistically significant differences were found between all genotypes studied (one asterisks indicates p = 0.05; two asterisks indicate p < 0.05). N of *w*^*1118*^ = 90, N of *park*^*1*^ = 90, N of *mul1*^*A6*^ = 90. **b** ROS level in the *Drosophila* brain (four asterisks indicate p < 0.01) (means ± SD). N of *w*^*1118*^ = 27, N of *park*^*1*^ = 25, N of *mul1*^*A6*^ = 28

Western blot analyses showed that the level of the main endogenous antioxidant superoxide dismutase (SOD1) was reduced in *mul1*^*A6*^ and *park*^*1*^ mutants by 55% and 45%, respectively, compared with the level in the control (Fig. 3a). Moreover, measurements of the fluorescence intensity associated with the free radical level in the whole *Drosophila* brain showed that the total ROS level was increased by 40% in both mutants (Fig. 3b).

### *mul1* and *park* mutations reduce autophagy

**Fig. 4:**
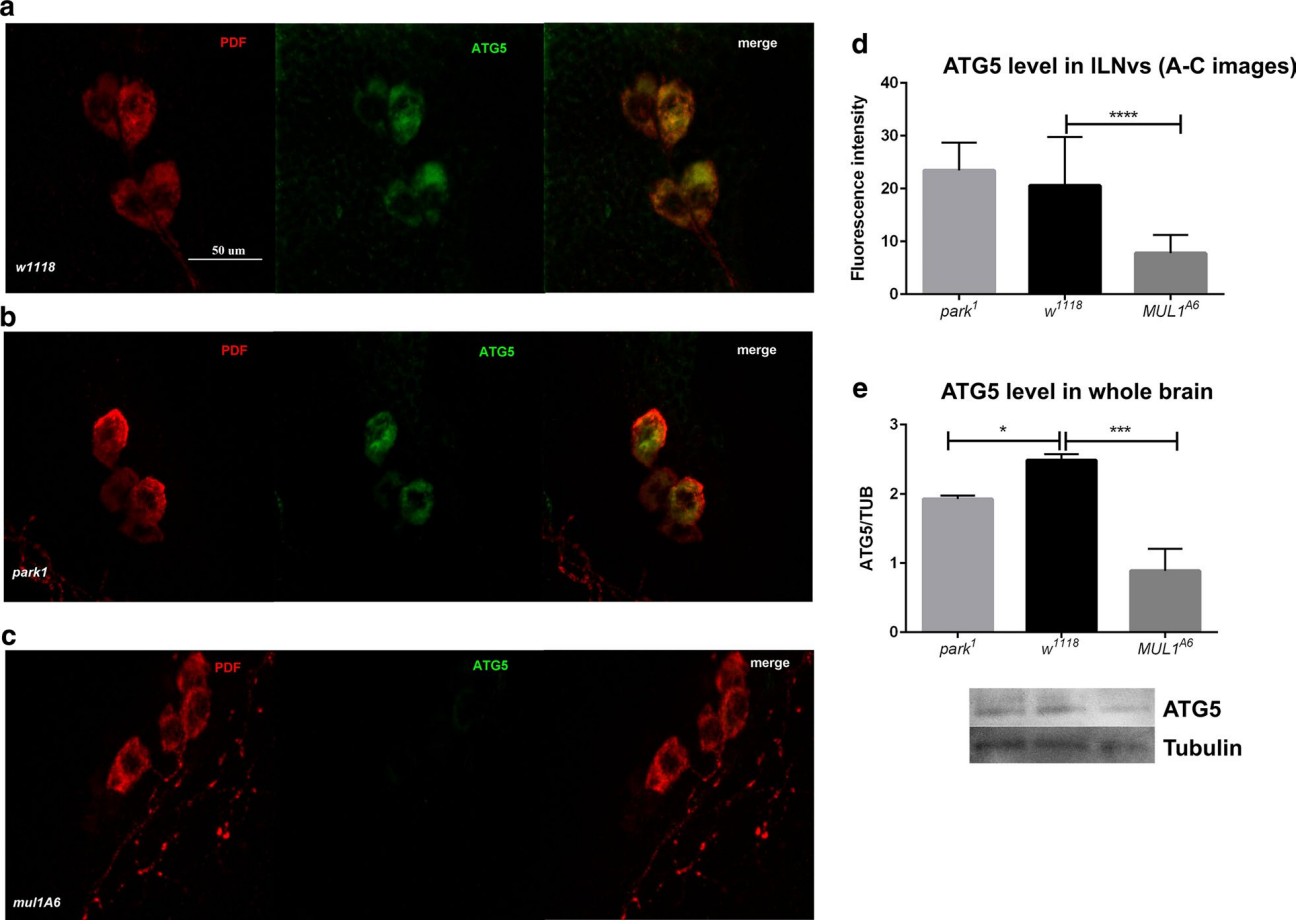
Effects of *mul1* and *park* mutations on ATG5 expression level in clock neurons and the *Drosophila* brain. **a–c** Co-localization of ATG5 and PDF in cell bodies of large ventral lateral clock neurons in *w*^*1118*^ (**a**), *park*^*1*^ (**b**) and *mul1*^*A6*^ (**c**). Scale bar: 50 µm. **d** ATG5 level in the l-LNvs (four asterisks indicate p < 0.01) (means ± SD). N of *w*^*1118*^ = 23, N of *park*^*1*^ = 21, N of *mul1*^*A6*^ = 15. **e** ATG5 in the whole *Drosophila* brain (means ± SD). Statistically significant differences were found between all genotypes studied (one asterisk indicates p < 0.05; three asterisks indicate p < 0.01). N of *w*^*1118*^ = 90, N of *park*^*1*^ = 90, N of *mul1*^*A6*^ = 90

Labelling of the clock neurons l-LNvs with antibodies against ATG5, an autophagy protein, and PDF, which is expressed in all l-LNvs, showed co-localization of both proteins in cell bodies of the l-LNvs (Fig. 4a–c). The analysis of fluorescence intensity associated with ATG5 expression in these neurons showed that in *mul1*^*A6*^ mutants, ATG5 abundance was decreased when compared with that in the control, while in *park*^*1*^ mutants, this difference was not statistically significant (Fig. 4d). The level of ATG5 in the whole brain in *mul1*^*A6*^ and *park*^*1*^ mutants was decreased by 70% and 20%, respectively, compared with that in the control (Fig. 4e). ATG5 showed expression almost everywhere in the brain, however, this signal was stronger in the l-LNvs and after the same settings of LSM it was possible to investigate the co-localization of ATG5 and PDF.

## Discussion

MUL1 and PARKIN proteins are responsible for mitophagy and seem to be involved in the development of Parkinson’s disease. Lack of these proteins leads to a reduced number of mitochondria and enlargement of their size [6, 9]. Our results showed that mutations in *mul1* and *park* increase ROS levels in the *Drosophila* brain, which origin from damaged mitochondria. While low levels of ROS are typical for normal cell metabolism, their excessive amounts cause oxidative stress and damage critical components of the cell by protein and lipid oxidation [20, 21]. Moreover, both mutations contribute to the reduction of SOD1, one of the main antioxidant proteins. SOD1 reduces free radical oxygen species, and its low level leads to the accumulation of ROS and the beginning of oxidative stress [22]. We also found changes of ATG5 level. The abundance of one of the core autophagy proteins, ATG5, was decreased in *mul1*^*A6*^ and *park*^*1*^ mutants, which suggests that autophagy is inhibited in both mutants. The failure of autophagy machinery to efficiently remove defective proteins or damaged organelles from the cytosol, increases the level of damaged cellular components [23] which accumulate inside the cell. On the other hand, it has been shown that higher oxidative stress causes an increase of autophagy to remove damaged proteins that may be a source of oxidative stress [24, 25]. However, when intracellular stress remains unresolved, prolonged autophagy up-regulation progresses into autophagy defect [26]. This finding explains why, despite of the increased ROS level, *mul1* and *park* mutants exhibited a decrease in the level of ATG5.

As previously mentioned, patients suffering from Parkinson’s disease exhibit sleep problems and have reduced lifespan. Our findings showed that in *mul1* and *park* models of PD, the lifespan is strongly reduced. The short lifespan may result from the reduced ATP levels. A low level of ATP was found in mutants of the *pink1* gene encoding PINK1, a kinase involved in mitophagy, which acts together with PARKIN. Moreover, mitochondrial morphology defects are similar in *pink1*, *park*^*1*^ and *mul1*^*A6*^ mutants. Therefore, the level of ATP can be concluded to be reduced with mutations of genes encoding mitochondrial ligases [27]. Furthermore, the results obtained by other authors suggest that inhibition of autophagy by reducing ATG9 (another autophagy protein) levels shortens the lifespan [28], and also ATG5 protein is responsible for. In addition to the shortened life time in mutants, we also observed increased locomotor activity and sleep disorders. The results of other authors confirm our results that the *park* mutation increases the daily locomotor activity of *Drosophila melanogaster* [29]. As mentioned previously, sleep is regulated by the circadian clock [30, 31] and in *Drosophila* is controlled via PDF-positive l-LNv neurons. They regulate total sleep as well as the rate of sleep onset [32]. Thus, sleep disorders may not results directly due to autophagy disruption and/or oxidative stress, but also indirectly due to circadian clock damage.

We also examined how the molecular circadian clock works in the mutant studied. The molecular mechanism of the circadian clock is based on the rhythmic expression of the core clock genes and proteins. First, we found differences in the daily expression profile of clock genes and PER protein in *park*^*1*^ and *mul1*^*A6*^ mutants. In *park*^*1*^ mutants, there was a shift of the peak of *per* and *tim* mRNAs to ZT16 and changes in the *clock* gene expression rhythm. In turn, in the *mul1*^*A6*^, mutants changes in the expression level of clock genes were observed, while the rhythms remained similar to those in the control. Despite the fact that the *per* gene in both mutants is rhythmically expressed, the rhythm of PER protein is completely disrupted. Between the maximum of *per* and *tim* mRNA levels and appearance of the maximum of their proteins is about 4 h shift (white control) due to post-transcriptional processing, translation and post-translational processes [33]. The differences between *per* transcription and PER translation may originate from abnormal activity of translational factors. Increased levels of ROS have been reported to affect the activity of the eIF2α translation factor [34]. In turn, the increased activity of eIF2α enhances the expression of various proteins, including PER, under stress conditions. High ROS levels may also degrade SLIMB, a core protein responsible for PER degradation in proteasomes [35]. Low SLIMB activity may result in the inhibition of PER degradation, thus increasing the PER level and changing its expression profile. ROS can also “reset” the circadian clock by modifying casein kinase 2 (CK2) [36]. CK2 phosphorylates PER and TIM and changes their levels, which may result in altered post-translational modification of PER [37]. ROS may also control the expression of clock genes. In mammals, a high level of ROS results in strong induction of clock gene expression [38]. ROS can also degrade transcription factors that regulate the expression of clock genes and proteins controlling the mRNA stability of clock proteins such as double-time kinase, NEMO kinase, VRILLE and PDP1ε [39–41]. A Shift in the transcription of clock genes between mutants and control may also be disturbed by changes in the level of other clock proteins: VRILLE and PDP1ε. These proteins act in the clock positive feedback loop, inhibiting and resuming the expression of the *clock* gene [42]. It is possible that the level and rhythmicity of these proteins is also altered, as in the case of PER protein, which in turn may cause the shift in the expression of *per* and t*im* clock genes in the mutants. However, more studies on other clock genes and proteins are needed. This shift can also be directly caused by the influence of oxidative stress and autophagy inhibition. We also found the co-localization of ATG5 and PDF in the somata of l-LNvs, which indicates that the clock proteins may also be degraded by autophagy. The l-LNvs are neurons in which the examined genes are expressed as well as PER at certain time of the day [43–45]. We observed that the level of ATG5 in these cells is reduced in both mutants, which may confirm the relationship between autophagy and the circadian clock. The difference between the results obtained in *park* and *mul1* is most likely due to the various functions of these proteins. Although PARKIN and MUL1 proteins are mitochondrial ligases involved in mitophagy, they also participate in other physiological processes. MUL1 participates in SUMOylation, i.e., the additional post-translational processing of proteins. SUMOylation can stabilize some proteins, such as a glucose transporter, on the cell membrane and is therefore necessary for their proper functioning [46]. SUMOylation can also stabilize tau and alpha-synuclein proteins, and the inhibition of this process may lead to the development of Parkinson’s disease [47]. SUMOylation also protects proteins against free radicals [48]. Inhibition of SUMOylation can thereby increase the sensitivity of proteins to ROS.

The main function of the circadian clock is to generate circadian rhythms in many gene expression in clock cells and transmit this information via eferential pathways to other tissues, thereby regulating the expression of genes and proteins of physiological, as well as various behavioral processes. An example of such behavioral process is the previously described sleep duration, as well as the rhythm of locomotor activity, for which *per* positive neurons are responsible for [49, 50]. Our results showed that *mul1* and *park* mutations cause the rhythm of locomotor activity period to be prolonged. In the case of *park* mutation, it has already been confirmed by another group of scientists who received a result similar to ours [29]. Mutations of the tested genes abolish the rhythm of the PER protein, and as a result the information about changing rhythms is erroneously transmitted to the effector tissues and disorders in the circadian rhythm of locomotor activity arise. This result is similar to the case in which *per* is mutated in many different forms [49, 50], which makes it even more convinced that *mul1* and *park* mutations lead to the circadian clock disruption.

## Conclusions

In conclusion, in the present study, we found that *mul1* and *park* mutations, which are involved in the development of Parkinson’s disease, disturb several processes in an organism, including circadian rhythms in behaviour and the molecular mechanism of the clock. This effect seems to originate from increased levels of free radicals and the inhibition of autophagy, which is important in circadian rhythm generation [51].

## Additional files

**Additional file 1: Table S1.** All raw data for the graphs presented in Figure 1. The sheets have numbered charts: Figure 1 - A, Figure 1 - B and Figure 1 - C respectively. In Figure 1 - A there are data for the survival curve. The “Day” column indicates in which day flies have died. The “Code” column means that the fly has died (1 - death). The “Group” column indicates the tested genotype. In Figure 1 - B there are data for the activity of the fruit fly: Total activity, sleep time during the day and at night, respectively. Columns marked “Minutes” indicate the time of activity / sleep of the fruit fly measured within 24 h. In Figure 1 - C there are results showing period of circadian rhythm of locomotor activity measured for 7 days under conditions of constant darkness.

**Additional file 2: Table S2.** All raw data for the graphs presented in Figure 1. The sheets have numbered charts: Figure 2 - A, Figure 2 – B, Figure 2 – C and Figure 2 -D respectively. In Figure 2 - A / B / C, there are presented results (RQ) for the expression of the *per*, *tim* and *clock* genes, respectively, at four time points. All data was averaged to the *rpl32*. Figures 2 - C show data for Western Blot analysis of PER protein. Data comes from densitometry, and has been averaged to load control - alpha tubulin

**Additional file 3: Table S3.** All raw data for the graphs presented in Figure 1. The sheets have numbered charts: Figure 3 – A and Figure 3 - B, respectively. Figure 3 - A shows the data for Western Blot analysis of the SOD1 protein. Results from densitometry have been averaged to load control - alpha tubulin. In Figure 3 - B there are data (fluorescence intensity measured using the ImageJ) showing the level of free radicals.

**Additional file 4: Table S4.** All raw data for the graphs presented in Figure 1. The sheets have numbered charts: Figure 4 – D and Figure 4 - E, respectively. Figure 4 - D shows the results (fluorescence intensity measured using the ImageJ) level of ATG5 protein in PDF-positive perycaryon of l-LNvs. In Figure 4 - E, the results of Western blot analysis of the ATG5 protein in the canine fruit fly are shown. Densitometry results were averaged to load control - alpha tubulin.

## Data Availability

The datasets obtained and analysed during the current study are available from the first and corresponding Authors on request.

## Abbreviations

PD: Parkinson’s disease
l-LNvs: large lateral neurons ventral
s-LNvs: small lateral neurons ventral
LNds: lateral neurons dorsal
DN: dorsal neurons
LPNs: lateral posterior neurons
PER: period
TIM: timeless
CLK: clock
CYC: cycle
DD: constant darkness
LD: light:dark
